# There is No Distinctive Gut Microbiota Signature in the Metabolic Syndrome: Contribution of Cardiovascular Disease Risk Factors and Associated Medication

**DOI:** 10.3390/microorganisms8030416

**Published:** 2020-03-15

**Authors:** Adrián Cortés-Martín, Carlos E. Iglesias-Aguirre, Amparo Meoro, María Victoria Selma, Juan Carlos Espín

**Affiliations:** 1 Laboratory of Food & Health, Research Group on Quality, Safety, and Bioactivity of Plant Foods, CEBAS-CSIC, Campus de Espinardo, 30100 Murcia, Spain; acortes@cebas.csic.es (A.C.-M.); ceiglesias@cebas.csic.es (C.E.I.-A.); mvselma@cebas.csic.es (M.V.S.); 2 Service of Endocrinology, Reina Sofía University Hospital, Avda. Intendente Jorge Palacios s/n, 30003 Murcia, Spain; amparoi.meoro@carm.es

**Keywords:** Gut microbiota, metabolic syndrome, obesity, cardiovascular risk, drug treatment, diabetes, dyslipidemia, hypertension, precision medicine

## Abstract

The gut microbiota (GM) has attracted attention as a new target to combat several diseases, including metabolic syndrome (MetS), a pathological condition with many factors (diabetes, obesity, dyslipidemia, hypertension, etc.) that increase cardiovascular disease (CVD) risk. However, the existence of a characteristic taxonomic signature associated with obesity-related metabolic dysfunctions is under debate. To investigate the contribution of the CVD risk factors and(or) their associated drug treatments in the composition and functionality of GM in MetS patients, we compared the GM of obese individuals (*n* = 69) vs. MetS patients (*n* = 50), as well as within patients, depending on their treatments. We also explored associations between medication, GM, clinical variables, endotoxemia, and short-chain fatty acids. Poly-drug treatments, conventional in MetS patients, prevented the accurate association between medication and GM profiles. Our results highlight the heterogeneity of taxonomic signatures in MetS patients, which mainly depend on the CVD risk factors. Hypertension and(or) its associated medication was the primary trait involved in the shaping of GM, with an overabundance of lipopolysaccharide-producing microbial groups from the Proteobacteria phylum. In the context of precision medicine, our results highlight that targeting GM to prevent and(or) treat MetS should consider MetS patients more individually, according to their CVD risk factors and associated medication.

## 1. Introduction

Fecal transplant experiments have demonstrated the causal role of the gut microbiota in disease [1,2,3,4,5,6,7]. These studies have indicated that both the alteration of the gut microbial ecosystem and its reversion are involved in the pathogenesis of multiple diseases such as obesity, depressive disorder, liver diseases, atherosclerosis, diabetes, inflammatory bowel diseases, asthma, Parkinson’s, Alzheimer’s, etc. [1,2,3,4,5,6,7,8].

Mounting evidence suggests that the modulation of the gut microbiota through different approaches such as specific lifestyle interventions (Mediterranean diet, physical activity, specific prebiotics, and probiotics, etc.), as well as fecal transplants, can be considered as promising adjuvants in the prevention or treatment of gut dysbiosis to counteract the onset or progression of chronic diseases [9,10,11].

The term ‘gut dysbiosis’ is commonly referred to as ‘imbalanced relative abundances’ of microbial groups, mainly inferred from 16S rRNA gene sequencing data. However, this description has drawn criticisms, and there is no full consensus regarding the term ‘gut dysbiosis’ [12,13]. The controversy mainly arises from the fact that a microbiome profile might be dysbiotic in some individuals and(or) be related to specific diseases, but similar profiles can be found in healthy subjects [14]. Besides, this paradox is also supported by the lack of a consensus definition of ‘healthy’ microbiome [14,15].

A paradigmatic example of the above controversy is the gut dysbiosis associated with metabolic dysfunctions such as obesity and metabolic syndrome (MetS) [16]. Whereas the presence of said dysbiosis has been commonly accepted in these conditions, there is an increasing debate regarding the presence of specific obesity-related microbial taxonomic signatures. For example, this is the case of the Firmicutes to Bacteroidetes ratio. While this ratio has been described to be higher in subjects with obesity-related dysfunctions vs. healthy lean individuals [17], other studies criticize this ratio and claim for the lack of a specific taxonomic signature associated with obesity [18]. A likely explanation for these controversies could lay on the variability of microbiome profiles depending on the geographical location. It has been reported that regional variation of microbiome signatures limits applications of specific ‘healthy gut microbiome’ reference ranges as well as microbiota-based disease models, which suggests that such models cannot be extrapolated [19]. In the same line, dysbiotic patterns have been reported to vary among studies depending on the locations [20,21].

Although there is no single definition for MetS [22], this pathological condition groups a constellation of metabolic factors that increase the risk of cardiovascular diseases (CVDs) (raised triglycerides, blood pressure, and glucose, reduced high-density lipoprotein cholesterol (HDLc), and central abdominal obesity) [23]. The gut microbiota composition and its potential role in MetS have been previously described [9,24,25,26,27,28], but the possible impact of drug treatments and(or) the specific cardiovascular risk factors in the composition and functionality of the gut microbiota from MetS patients have not been addressed. Similarly, the microbiota in animal MetS models is well characterized [26,29]. However, the induction of MetS in these models is different, and the medication is not present [30]. Therefore, the interaction between microbiota and specific MetS treatments and(or) their medication in these models is also unknown. Overall, the impact of some drugs such as antibiotic treatments on the gut microbiota is widely known, but the effect of pharmacological therapy commonly used in the management of patients with metabolic diseases is still poorly understood [31].

We hypothesize that the microbiota signature in MetS is heterogeneous and depends not only on the regional variability or other conditions such as lifestyle but could be critically affected by the primary condition (diabetes, hypertension, and dyslipidemia) of the patients and(or) the associated medication. In the present pilot study, we aimed to i) compare the gut microbiota of obese subjects vs. (poly)-medicated obese MetS patients at high risk of CVD but with no established disease, ii) explore the possible contribution of the main trait (diabetes, hypertension, and dyslipidemia) or the associated drug treatments (anti-hypertensive, anti-diabetic, and lipid-lowering drugs) on the gut microbiota signature of these patients, and iii) investigate potential associations among medication, gut microbiota, clinical variables, endotoxemia, and short-chain fatty acids (SCFAs).

## 2. Materials and Methods

### 2.1. Study Subjects

This study followed the ethical guidelines outlined in the Helsinki Declaration of 1975 and its amendments. The Clinical Ethics Committee at Reina Sofía University Hospital (Murcia, Spain) and the Spanish National Research Council’s Bioethics Committee (Madrid, Spain) (reference AGL2015-64124-R) approved this protocol. The trial was registered at clinicaltrials.gov as NCT04075032. The study was fully explained to the patients who gave their written informed consent before participating. Eligible participants were those patients with no established cardiovascular disease, over 18 years, and under secondary pharmacological prevention for metabolic syndrome (MetS) (i.e., under drug therapy when a healthy lifestyle is not enough to prevent the imbalance of cardiovascular risk factors). MetS was diagnosed according to the International Diabetes Federation [23], i.e., waist circumference >94/80 cm (Caucasian males/females), or BMI > 30 kg/m^2^, plus any two of the following four factors: i) raised triglycerides (≥150 mg/dL) or specific treatment for this lipid abnormality, ii) reduced HDLc (<40 mg/dL in males and <50 mg/dL in females) or specific treatment for this lipid abnormality, iii) raised blood pressure (BP) (systolic BP ≥ 130 or diastolic BP ≥ 85 mmHg) or treatment for hypertension, and iv) raised fasting plasma glucose (≥100 mg/dL) or previously diagnosed type 2 diabetes. Exclusion criteria were: previously diagnosed cardiovascular disease (coronary artery disease, peripheral artery disease, stroke, thromboembolic disease, etc.), pregnancy, lactation, previous gastrointestinal surgery, and the use of antibiotics or nutraceuticals within 1 month before the study. The patients were also requested to provide a food questionnaire with their specific diet followed for three days before the collection of samples. For comparative purposes, samples from obese subjects (OB) (i.e., volunteers with BMI > 30 kg/m^2^) from previous trials (*n* = 69), with no diagnosed disease, were also used in the present study [32].

### 2.2. Sampling Procedure

Fasting blood and feces samples were collected in the morning. The feces were stored at −80 °C until further analysis. Blood samples were collected in two vacutainer tubes, one of them with EDTA to obtain the whole blood and plasma after centrifuging at 2000 g at 4 °C for 10 min, while the other one, without anticoagulant, to obtain serum samples, after centrifuging under the same conditions. All samples were aliquoted and frozen at −80 °C until analysis.

### 2.3. Determination of Serobiochemical Variables and Lipopolysaccharide-Binding Protein (LBP)

Serobiochemical variables including glucose, bilirubin, protein, gamma-glutamyl transferase (GGT), alanine aminotransferase (ALT), aspartate aminotransferase (AST), alkaline phosphatase (ALP), lactate dehydrogenase (LDH), total cholesterol (Tchol), LDL cholesterol (LDLc), HDLc, triglycerides, calcium, sodium, phosphorus, potassium, chlorine, albumin, urea, and creatinine were measured in serum samples using automated biochemical auto-analyzers (Advia Systems, Siemens Healthcare Diagnostic Inc., Deerfield, IL, USA). Insulin was measured with the IMMULITE 2000 analyzer (DPC, LA, USA), and insulin resistance was calculated with the Homeostatic Model Assessment for Insulin Resistance (HOMA-IR). Whole blood was used to determine red and white cell series by an automated hematological analyzer (LH 780; Beckman Coulter, Fullerton, CA, USA). We determined plasma lipopolysaccharide-binding protein (LBP), a surrogate marker of metabolic endotoxemia, since plasma lipopolyshaccharide (LPS) determination shows several limitations, mainly due to the presence of endogenous inhibitors [33]. LBP was determined using a commercial ELISA kit (HycultBiotech, Uden, The Netherlands) as previously reported [34]. All samples were analyzed in triplicate, with intra- and inter-assay coefficients of variations (CVs) <10% for all parameters.

### 2.4. Gut Microbiota Analysis

Bacterial DNA was extracted from fecal samples using the NucleoSpin^®^ Tissue DNA Purification Kit (Macherey-Nagel, Germany), following the manufacturer’s instructions. Gut microbiota composition and diversity were determined by the V3-V4 variable region of the 16S rRNA gene sequencing, following Illumina protocols (Illumina Inc., San Diego, CA, USA). Libraries were sequenced on a MiSeq-Illumina platform (FISABIO sequencing service, Spain) using a MiSeq Reagent kit v3 (MS-102-3001) with a read length of 2 × 300 bp paired-end run. Data processing (quality assessment and removal of chimeric sequences), sequence alignment, 16S rRNA gene sequence clustering, and alpha-diversity indexes (Shannon and Chao1 indexes) of gut microbiota were carried out as previously described [32]. The estimation of the samples’ alpha-diversity indexes (Chao1 and Shannon) was based on a randomly selected 30,992 reads per sample. Potential bacterial functions were identified by Phylogenetic Investigation of Communities by Reconstruction of Unobserved States (PICRUSt) v0.9.0 [35].

### 2.5. Short-Chain Fatty Acids (SCFAs) Determination

SCFAs were determined in stool samples, as previously reported, with some modifications [36]. Fecal samples were diluted (1:1) with 0.9% NaCl in water and centrifuged. The supernatants (400 μL) were acidified with 40 µL of 5% *o*-phosphoric acid, vortexed, and extracted with 400 µL of methyl *t*-butyl ether. Samples were vortexed for 3 min and centrifuged at 10,000 g for 10 min at 4 °C. The organic phase was collected, and 5 µL of 10 mM 4-methyl valeric acid was added as an internal standard. The sample was injected in a gas chromatography-mass spectrometer (GC-MS) (Agilent 7890A coupled with an Agilent 5975C mass selective detector) (Agilent Technologies, Santa Clara, CA, USA) using a fused silica capillary column DB-WAXetr (30 m, 0.25-mm id, 0.25-mm film thickness; Agilent), and helium as carrier gas at 1 mL/min. The column temperature was initially set at 90 ºC, increasing to 150 °C at 15 °C /min, to 170 °C at 5 °C /min, and finally to 250 °C at 20 °C /min, keeping this temperature for 2 min. The detector was operated in electron impact ionization mode (electron energy 70 eV), scanning the 30-250 *m*/*z* range. The temperatures of the quadrupole, interface, and ion source were 150, 289, and 230 °C, respectively. Standard compounds were used to identify SCFAs (acetic, propionic, isobutyric, butyric, isovaleric, and valeric acids).

### 2.6. Statistical Analysis

SPSS Software version 26.0 (SPSS Inc., Chicago, IL, USA) was used for data analysis. Data normality was evaluated with the Shapiro–Wilk test. Comparisons of serobiochemical variables, LBP, SCFAs, and bacterial groups between two clusters (obese subjects vs. MetS patients, type of medication, etc.) were conducted using independent sample *t*-test or the Mann–Whitney *U* test for normal or non-normally distributed data, respectively. When more than two groups were compared, analyses of variance (ANOVA) and Bonferroni *t*-test or the Kruskal–Wallis followed by Dunn’s test were used for normally and non-normally distributed data, respectively. Sex and age were considered as possible covariates in all the analyses. Linear discriminant analysis effect size (LEfSe) method was performed using the online interface Galaxy (available online: http://huttenhower.sph.harvard.edu/galaxy/root) to differentiate the specific bacterial taxa in relative abundance between two groups (obese subjects vs. MetS patients) and to identify potential taxa and microbial functions associated with the drug treatments within MetS patients (consumers of anti-hypertensive drugs vs. non-consumers, etc.). Correlations between parametric variables were carried out with the Pearson’s correlation, while non-parametric variables were analyzed with the Spearman’s correlation. Sigma Plot 13.0 (Systat Software, San Jose, CA, USA) was used to perform plots of data. Statistical significance was set at * *p* < 0.05, ** *p* < 0.01, and *** *p* < 0.001.

## 3. Results

### 3.1. Demographic Characteristics and Laboratory Values of the Population Study

A total of 69 eligible metabolic syndrome patients were contacted, and 50 accepted to provide both plasma and stool samples. Table 1 shows the main demographic characteristics and laboratory values of the MetS patients (24 women and 26 men) participating in the study. Sex-specific differences were found for HDLc, GGT, insulin, HOMA-IR, creatinine, and LBP. All the patients were under drug therapy to prevent cardiovascular events. The questionnaires provided by the patients were quite similar (results not shown) and matched with the lifestyle recommendations for MetS management, including adherence to the Mediterranean diet, smoking cessation, moderate physical activity, etc. [37]. Regarding the obese group, the main demographic characteristics and plasma LBP values are shown in Table S1. No significant differences were observed in BMI, age, and LBP values between women (*n* = 37) and men (*n* = 32) (Table S1).

For further comparative purposes, and taking into account the heterogeneity of the drug treatments, we grouped the patients according to the drugs that they consumed, indicative of their associated CVD risk factors (i.e., hypertension, dyslipidemia, and diabetes). The patients were poly-medicated with three primary types of drug families (Table S2), i.e., oral anti-diabetics (AD) (mainly metformin, but also with different combinations of glucusorics and insulin sensitizers), lipid-lowering drugs (LL) (mainly statins, but also fibrates and ezetimibe), and anti-hypertensive drugs (HP) (mainly angiotensin II receptor blockers, angiotensin-converting enzyme inhibitors, as well as diuretics, calcium channel blockers, and β-1 adrenergic blockers).

Consequently, we established the following groups for further comparisons: (i) MetS-all (all the patients); (ii) MetS-AD, diabetic patients that consumed oral AD; MetS-LL, patients with dyslipidemia that consumed LL drugs; and (iii) MetS-HP, those patients with diagnosed hypertension and consuming anti-hypertensive drugs. We acknowledge that some patients were present in more than one group because it is inherent to MetS the existence of at least two of these CVD risk factors. Therefore, the comparisons were established between OB subjects (OB) and MetS patients as well as within MetS, according to their primary drug treatments associated with their CVD risk factors (e.g., MetS-HP vs. MetS-HP non-treated, etc.).

### 3.2. Alpha-diversity Indexes of the Gut Microbiota

Diversity (Shannon index) was not different between OB and MetS patients (results not shown), whereas richness (Chao index) was higher in MetS patients vs. OB, although no differences were observed within MetS patients depending on their drug treatments (Figure S1).

### 3.3. Comparison of Taxonomic Signatures in Obese Subjects and MetS Patients

**Figure 1:**
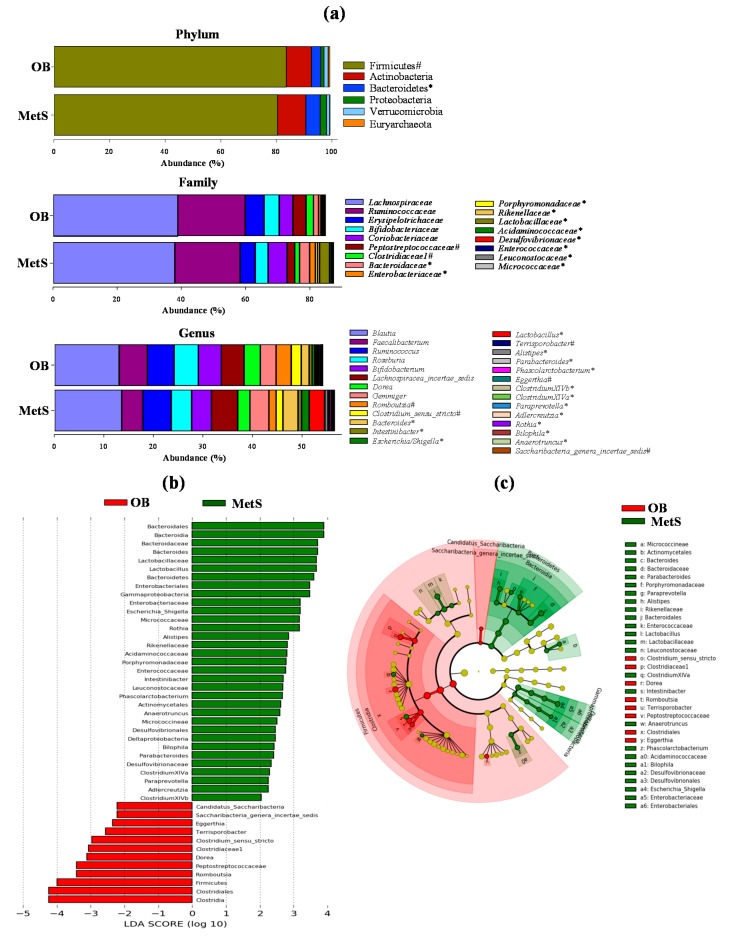
(**a**) Microbial taxonomic composition in fecal samples from obese (OB) and metabolic syndrome (MetS) participants, showing the mean abundance (%) at phylum, family, and genus levels. ^#^ Higher abundance in OB; * higher abundance in MetS. (**b**) Differently abundant bacterial communities between OB and MetS were identified using linear discriminant analysis (LDA) combined with effect size (LEfSe) algorithm. The taxa shown have a value of LDA score (log10) above 2.0. (**c**) Cladogram, derived from LEfSe analysis of differential gut microbial taxa, is represented by rings with phyla in the outermost ring and genera in the innermost ring. The nodes indicate the abundance of the microorganism. Red and green nodes represent taxa significantly (*p* < 0.05) overabundant in OB and MetS, respectively, while yellow nodes indicate taxa that were not differentially abundant (*p* > 0.05).

**Figure 2:**
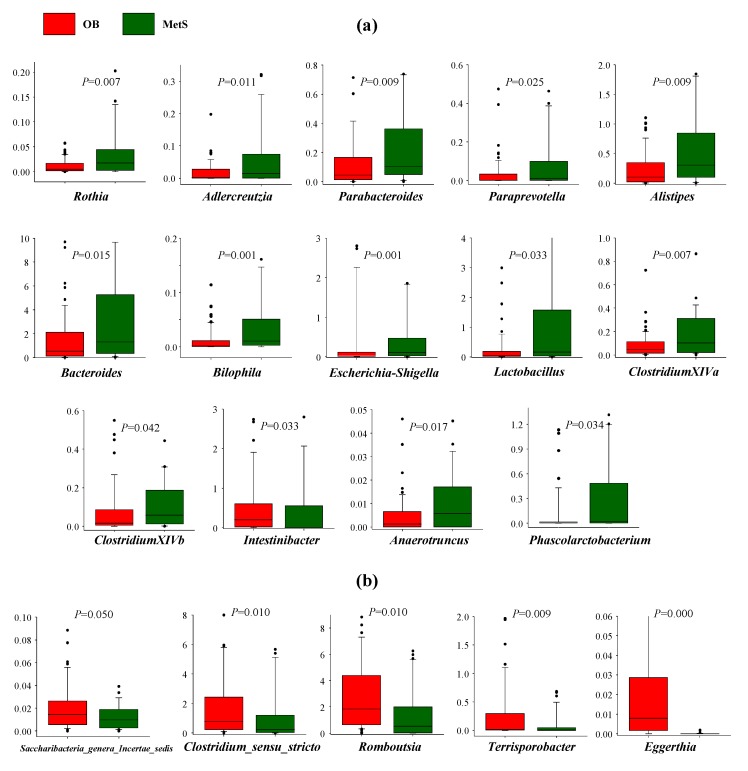
Genera significantly (*p* < 0.05) overabundant in (**a**) MetS vs. OB, and (**b**) overabundant in OB vs. MetS. OB, red bars; MetS, green bars. The comparison was performed using the Mann–Whitney Rank Sum Test.

At the phylum level, Firmicutes predominated in OB, whereas Bacteroidetes were more abundant in MetS patients vs. OB. The families *Bacteriodaceae*, *Enterobacteriaceae*, *Porphyromonadaceae*, *Rikenellaceae*, *Lactobacillaceae*, *Acidaminococcaceae*, *Desulfovibrionaceae*, *Enterococcaceae*, *Leuconostocaceae,* and *Micrococcaceae* were more abundant in MetS than in OB, whereas the families *Peptostreptococcaceae* and *Clostridiaceae* predominated in OB vs. MetS patients (Figure 1a). Linear discriminant analysis (LDA) effect size (LEfSe) (Figure 1b) and cladogram (Figure 1c) show the taxonomic representation of statistically and biologically consistent differences between OB and MetS patients, highlighting the predominance of the phylum Firmicutes and the class Clostridia in OB vs. MetS, and the phylum Bacteroidetes, and the classes Bacteroidia, Gammaproteobacteria, and Deltaproteobacteria in Mets vs. OB, as well as confirming the rest of differences observed at genus, family, order, class, and phylum levels. No sex-specific differences were found within OB nor in the comparison OB vs MetS. Figure 2 shows the higher relative abundance of representative bacterial groups at the genus level in MetS vs. OB (Figure 2a) and OB vs. MetS (Figure 2b).

**Figure 3:**
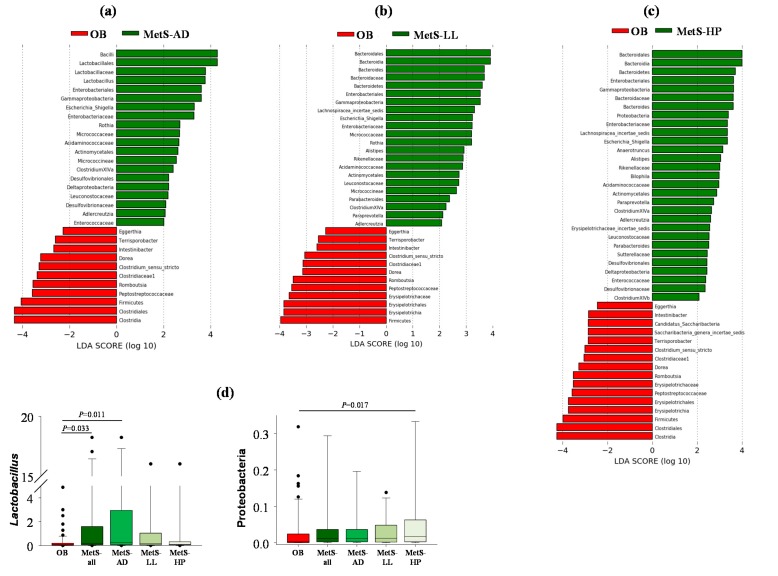
Contribution of drug therapy to the differently abundant genera (*p* < 0.05) in OB vs. MetS patients. (**a**) OB vs. AD-consuming MetS patients (MetS-AD); (**b**) OB vs. LL-consuming MetS patients (MetS-LL); (**c**) OB vs. HP-consuming MetS patients (MetS-HP); (**d**) *Lactobacillus* and Proteobacteria in MetS-AD and MetS-HP patients, respectively, as microbial groups significantly associated with drug treatments (or their corresponding linked CVD risk factor) in the comparison of OB vs. MetS. AD, oral anti-diabetics; LL, lipid-lowering drugs; HP, anti-hypertensive drugs. The comparison was performed using ANOVA on Ranks and Dunn’s test.

Taking into account the above results, we next explored the potential contribution of the primary CVD risk factors (diabetes, dyslipidemia, and hypertension, and(or) their associated drug treatments) in the different taxonomic signatures observed between OB and MetS patients. As in the case of OB, no sex-specific differences in the gut microbiota were found within MetS patients. Figure 3a–c show statistically significant differences in the taxonomic signature of OB vs. MetS-AD, OB vs. MetS-LL, and OB vs. MetS-HP patients, respectively. However, a further statistical analysis comparing all groups at once to deepen the specific contribution of each medication (or CVD risk factor) on microbial gut composition revealed only two microbial groups, i.e., *Lactobacillus* genus and Proteobacteria phylum that were significantly enriched in MetS-AD and MetS-HP patients, respectively, vs. OB (Figure 3d).

### 3.4. Firmicutes to Bacteroidetes ratio (F/B) in OB and MetS Patients

**Figure 4:**
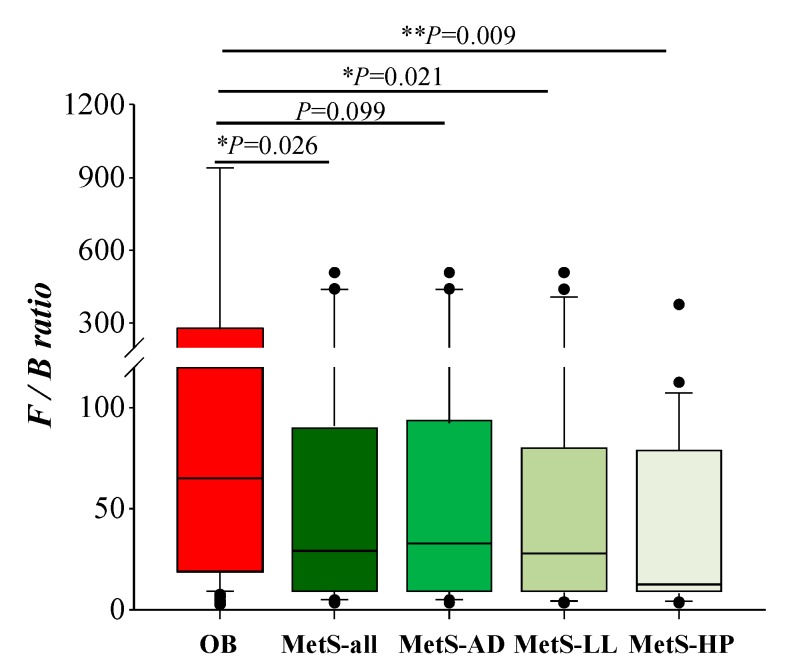
Comparison of the Firmicutes to Bacteroidetes ratio (F/B) in OB vs. MetS patients, as well within MetS, depending on the drug therapy. AD, oral anti-diabetics; LL, lipid-lowering drugs; HP, anti-hypertensive drugs. The comparison was performed using ANOVA on Ranks and Dunn’s test.

Continuing with the comparison between OB and MetS, and the possible specific contribution of drug treatments and(or) their associated CVD risk factors, we analyzed differences in the F/B ratio. As shown in Figure 4, F/B was higher in OB vs. MetS patients. However, this difference did not reach statistical significance in the comparison of OB vs. MetS-AD, while the difference between OB and MetS-HP patients reached the highest significance (Figure 4). Besides, F/B was not significantly different within MetS patients, according to their drug treatments (Figure 4).

### 3.5. Metabolic Function Prediction in OB and MetS Microbiota Profiles

LEfSe analysis, performed on metabolic functions inferred by PICRUSt analysis, showed statistically significant enrichment of carbohydrate metabolism in MetS vs. OB (Figure S2a). The comparison of OB vs. MetS-AD, -LL, and -HP patients (Figure S2b, S2c, and S2d, respectively) confirmed the enrichment of the aminosugar metabolism pathway in MetS patients vs. OB (Figure S2a). The comparison of OB vs. MetS-AD revealed the additional enrichment of the galactose metabolism (Figure S2b), whereas the comparison of OB vs. MetS-LL also revealed the enrichment of the cytochrome P450 pathway (Figure S2c), and finally, the comparison of OB vs. MetS-HP showed the enrichment of the pathway related to membrane and intracellular structural molecules (Figure S2d). Within MetS, no differences were observed between MetS-LL and MetS non-LL patients, while the pathways mainly related to cell motility processes predominated in MetS non-AD vs. MetS-AD patients (Figure S2e), and the k3 KEGG pathway dealing with membrane transport predominated in MetS non-HP vs. MetS-HP patients (Figure S2f).

### 3.6. Taxonomic Signatures in MetS Patients Grouped by Pharmacological Treatments

**Figure 5:**
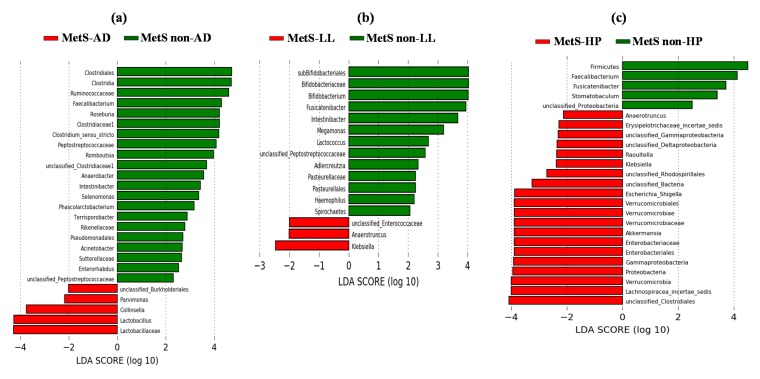
Differently abundant bacterial groups within MetS patients, depending on their therapy, using LEfSe analysis. (**a**) AD, oral anti-diabetics; (**b**) LL, lipid-lowering drugs; (**c**) HP, anti-hypertensive drugs.

After comparing taxonomic signatures between OB and MetS patients, we next investigated possible differences in the gut microbial profile within MetS patients to evaluate the potential contribution of the primary CVD risk factor and(or) their associated drug treatments in the gut microbiota profiles. Firstly, we did not identify significant associations between lifestyle and microbial groups within MetS patients (results not shown). On the search of specific microbial groups, potential biomarkers of each drug treatment, LEfSe analysis showed the enrichment of *Parvimonas*, *Collinsella*, *Lactobacillus*, and unclassified Burkholderiales in MetS-AD (Figure 5a), unclassified *Enterococcaceae* in MetS-LL (Figure 5b), and the MetS-HP group showed the specific enrichment of several groups, mainly Proteobacteria, *Lachnospiracea incertae sedis*, *Enterobacteriaceae*, Verrucomicrobia (mainly represented by *Akkermansia*), *Escherichia-Shigella*, *Raoultella*, and unclassified Clostridiales and Rhodospirillales (Figure 5c). The enrichment of the Proteobacteria phylum, with LPS-producing members, in MetS-HP patients (Figure 3c,d; Figure 5c) prompted us to compare plasma LBP levels within MetS patients, according to their medication.

### 3.7. Hypertension Correlates with Higher Plasma LBP Values in MetS

**Figure 6:**
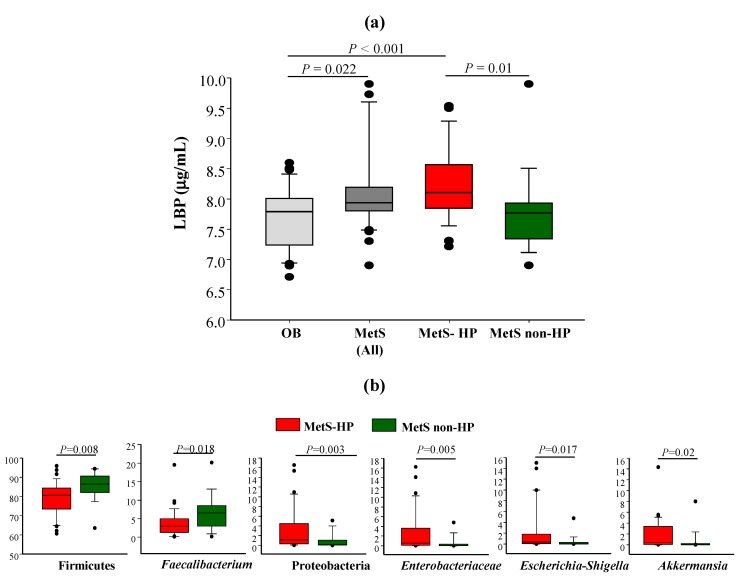
(**a**) Comparison of the plasma lipopolysaccharide-binding protein (LBP) concentration in OB, all MetS patients, MetS-HP, and MetS non-HP. The comparison was performed using ANOVA on Ranks and Dunn’s test. (**b**) Genera potentially involved in endotoxemia-related processes that were significantly different in MetS-HP vs. MetS non-HP patients.

Plasma LBP values were significantly higher in MetS patients vs. OB (Figure 6a). Hypertensive patients mainly contributed to this difference since LBP values were only different between OB and MetS-HP but not between OB and MetS non-HP (Figure 6a). Besides, no significantly different LBP values were observed within MetS-AD, MetS-LL, and MetS-HP patients, possibly due to the presence of some hypertensive patients in the AD and LL groups, as previously commented in Section 3.1 (results not shown).

We next compared the relative abundance of significantly different microbial groups (Figure 5c), potentially involved in protective or triggering endotoxemia-related processes, in MetS-HP vs. MetS non-HP patients (Figure 6b), as well as their association with plasma LBP values. Spearman’s correlations revealed positive correlations between plasma LBP values and significantly different bacterial groups at phylum, class, order, family, and genus levels, such as Proteobacteria (r = 0.502, *p* = 0.002), Gammaproteobacteria (r = 0.389, *p* = 0.019), Enterobacteriales-*Enterobacteriaceae* (r = 0.375, *p* = 0.024), and *Escherichia-Shigella* (r = 0.401, *p* = 0.015). No significant associations were observed between LBP and the rest of the groups, including Firmicutes, *Faecalibacterium,* and Verrucomicrobia (*Akkermansia*).

### 3.8. Lower SCFA Production Correlates with Lipid-lowering (LL) Treatments

Finally, we analyzed the SCFA profile (acetic, propionic, isobutyric, butyric, isovaleric, and valeric acids) in MetS patients to explore possible differences, according to their drug treatments. We only observed significantly lower levels of butyric and acetic acids in MetS-LL vs. MetS non-LL patients (Figure S3). The correlation of microbial groups significantly different between MetS-LL and MetS non-LL (Figure 5c) with SCFAs revealed that *Fusicatenibacter* positively correlated with higher levels of butyric and acetic acids (r = 0.486, *p* = 0.004, and r = 0.452, *p* = 0.004, respectively) and *Anaerotruncus* and butyric acid (r = 0.421, *p* = 0.012) in MetS non-LL patients.

## 4. Discussion

There is no full consensus regarding the presence of specific microbial taxonomic signatures in obesity (OB)-associated dysfunctions. Recently, Zeng et al. [38] proposed discriminant gut microbiota profiles in a Chinese population as possible biomarkers to predict obesity-related metabolic dysfunctions. However, Ahmad et al. [39] did not find the same profiles in obese Pakistani subjects with type-2 diabetes vs. lean individuals. Besides, these results were also different from those reported for an Indian population [40], which could be related to geographical variability [19] and other factors not well-defined yet [20,40]. In the same line, the Firmicutes to Bacteroidetes ratio (F/B) is also under debate as a possible biomarker of obesity and related dysfunctions [17,18,24]. Overall, these and other reports suggest that many confounding variables could prevent the existence of a unique taxonomic signature as a standard feature for obesity and associated comorbidities. The complexity of this topic increases in obese subjects with a cluster of CVD risk factors, such as in MetS.

In the present study, the profile of the potential gut microbiota biomarkers of OB-related dysfunctions previously proposed by Zeng et al. [38] was improved in MetS patients vs. OB. These biomarkers included *Bacteroides*, *Parabacteroides*, *Clostridium XIVa*, *Alistipes, Dorea*, *Romboutsia*, and *Clostridium sensu stricto*. Besides, the F/B ratio was also improved in MetS patients vs. OB. Overall, these results might indicate a paradoxically ‘healthier’ gut microbiota profile in MetS patients vs. OB. However, the same comparison, but clustering the patients as a function of their drug treatments (AD, LL, or HP), revealed distinctive differences between groups with enrichment of Proteobacteria in hypertensive patients and *Lactobacillus* in MetS patients with diabetes (MetS-AD) vs. OB. Besides, the F/B ratio was not significantly different in MetS-AD patients vs. OB, while the highest significant difference was observed in the MetS-HP group. Therefore, these results illustrate the heterogeneity of the gut microbial profile in MetS patients as a function of their drug treatments and(or) primary CVD risk factors (diabetes, hypertension, dyslipidemia).

In contrast with our results, Haro et al. [9] reported a lower F/B ratio in OB vs. MetS patients, as well as other dissimilarities, including enrichment of Actinobacteria in MetS, but not Proteobacteria vs. OB. Two main factors could explain these differences. Firstly, those patients with MetS features had established coronary artery disease (CAD) [9]. In this regard, recent evidence describes the alteration of gut microbiota in established cardiovascular disease [41]. Besides, myocardial infarction has been reported to modulate the blood microbiota, mediated by the increase of intestinal bacterial translocation [42]. Secondly, Haro et al. [9] did not address possible differences between OB and MetS, depending on the associated drug treatments. In our study, the analysis of patients as a single group or differentiating according to medication did not yield the same results.

The effect of drug treatments on the gut microbiota is still poorly understood. Not being able to separate the ‘disease’ variable (host condition) from its pharmacological treatment (sometimes poly-medication as in the case of MetS) avoids discovering that specific drug effect. Recent evidence shows, mainly in animal models, a dysbiotic-prone effect of statins through unpaired bile acid production that alters the gut microbiota [43,44]. Moreover, the gut dysbiosis in diabetic patients has been described as well as how anti-diabetic drugs such as metformin can modulate the gut microbiota, contributing to metformin glucose-lowering effects [45]. However, other studies have reported that the modulation of the microbiota by metformin treatment could contribute to its gastrointestinal adverse effects [46] or even promote dysbiosis [47] as described in healthy patients.

The poly-pharmaceutical treatment of the MetS patients participating in the present study leads us to think that the primary variable conditioning the microbiota in MetS lay in the cardiovascular risk factors. This is the case of patients with anti-hypertensive treatment, which included in many cases a complex of different angiotensin II receptor blockers, angiotensin-converting enzyme inhibitors, diuretics, calcium channel blockers, and β-1 adrenergic blockers. Although there are no human studies that specifically address the possible effect of this mixture of anti-hypertensive drugs on the gut microbiota [48], a recent study shows that the angiotensin receptor blocker losartan exerted anti-hypertensive effects, at least partially, by modulating the gut microbiota of spontaneously hypertensive rats [49]. Nevertheless, we speculate that the significant structural variability of anti-hypertensive drugs might prevent the elucidation of common mechanisms of action (either direct or indirect) on the gut microbiota.

The correlation between gut dysbiosis and hypertension has been previously established [50]. We show here that the gut microbiota of hypertensive patients was enriched in Proteobacteria, including the LPS-producing microbial groups *Enterobacteriaceae* and *Escherichia-Shigella*, while the content of Firmicutes and *Faecalibacterium* was higher in non-hypertensive patients. In agreement with our results, González-Sarrías et al. [34] reported the inverse association between plasma LBP values and *Faecalibacterium* in obese subjects. In the same line, Wang et al. [51] described higher plasma LPS values in preeclampsia associated with the depletion of Firmicutes and enrichment of Proteobacteria in patients vs. controls. Similarly, Chang et al. [52] have also shown the correlation between LPS production and Proteobacteria, including the genus *Escherichia-Shigella*, also in preeclampsia patients. Therefore, our results show a possible microbial LPS-producing link between hypertension in MetS patients and preeclampsia, a pregnancy-specific, complex, and multisystem hypertensive disorder [53]. Interestingly, Li et al. [54] established the relationship between gut dysbiosis and hypertension in a Chinese cohort. These authors provided relevant evidence for the causal role of gut microbiota dysbiosis as a key factor for blood pressure changes. In agreement with our results, this report linked the increase of LPS-producing bacteria, including Proteobacteria, with hypertension [54].

SCFAs are microbial metabolites that illustrate the link between diet, gut microbiota, and health [25]. SCFAs such as butyrate, acetate, and propionate have been associated with the mechanisms through which gut microbiota could influence blood pressure [55]. In addition to cross-sectional associations between SCFA production and hypertension, the causal protective role of SCFAs such as propionate (attenuation of cardiac hypertrophy, fibrosis, vascular dysfunction, and hypertension) has been demonstrated in animal models of hypertensive cardiovascular damage [56]. Although lower fecal levels of butyric and valeric acids have been described in patients with preeclampsia [52], we did not find differential SCFAs values in MetS-HP patients, which was in agreement with Sun et al. [48] who did not find any association between SCFA production and hypertension. SCFAs have also been proposed as microbial metabolites with a promising therapeutic role in mitigating long-standing dyslipidemia [57]. In this regard, we observed a positive correlation between butyrate and acetate levels and *Fusicatenibacter,* specifically in MetS non-LL, which agrees with previous studies in patients with cirrhotic dysbiosis [58].

The supplementation with *Akkermansia muciniphila* has been reported to improve several metabolic parameters in humans with insulin resistance without altering the gut microbiota ecology [59]. Paradoxically, *Akkermansia* was enriched in MetS-HP patients vs. MetS non-HP patients. Qin et al. [60] also observed the overabundance of *Akkermansia* in samples from diabetic patients. However, although the possible protective effects of *A. muciniphila* against metabolic diseases were already known [61], they did not provide a likely explanation for that apparent paradox [60]. Recently, Salguero et al. [62] reported Gram-negative dysbiosis in type-2 diabetic patients with chronic kidney disease vs. controls, which was associated with metabolic endotoxemia and inflammation, and also identified overabundance of Proteobacteria, Fusobacteria, and Verrucomicrobia (*Akkermansia*) in these patients. These authors did not provide any plausible explanation for this finding. Since the causality of *Akkermansia* as a protective group against the gut barrier alteration and subsequent metabolic dysfunction has been demonstrated [59], we believe its coexistence with Proteobacteria and other LPS-producing groups deserves further research and provide an additional issue to the lack of consensus regarding the term ‘gut dysbiosis’.

We acknowledge that our design does not allow distinguishing whether the gut microbiota from MetS patients is mainly affected by their primary traits (hypertension, diabetes, and(or) dyslipidemia) and(or) by the associated drug treatments. Besides, the sample size of this pilot study prevented further subgrouping of patients from making a definite difference within specific treatments. We initially also aimed to include a group of volunteers with diagnosed MetS but without medication (i.e., under primary prevention with lifestyle interventions). However, there was an insufficient sample size without any medication to compare groups robustly, and they were left out from the analyses. Besides, discriminating cause-and-effect relationship is challenging since 16S rRNA gene sequencing captures microbial profiles at a particular moment in time. Therefore, it is difficult to ascertain whether an altered microbial profile is a cause or a result of the disease.

## 5. Conclusions

Overall, the cluster of CVD risk factors, and the associated poly-pharmaceutical treatment in MetS, illustrates the lack of unique taxonomic signature for metabolic dysfunctions. The heterogeneity arises from both the presence of different risk factors within the same population of MetS patients under study and by the miscellanea of drug treatments associated with MetS traits as possible contributors to the microbial signature of the patients [31]. In the present pilot study, our results suggest that there is no distinctive taxonomic signature in MetS, but mainly depends on the CVD risk factors. Unfortunately, drug treatments are unavoidably present in patients under secondary prevention of MetS, and, in agreement with Sun et al. [48], we could not accurately identify the possible contribution, if any, of the medication to the gut microbiota profile of the patients. In this regard, we cannot discard possible additive or synergistic or counteracting effects between the poly-medication and MetS traits in the modulation of the gut microbiota. However, this is a challenging task that remains to be investigated.

This study lays the groundwork for considering patients with metabolic syndrome more individually, according to their cardiovascular risk factors and associated medication. This is especially important if it is intended to modulate the gut microbiota as a possible therapeutic objective for the prevention and treatment of the metabolic syndrome.

## Figures and Tables

**Table 1:** Demographic and laboratory values of the metabolic syndrome (MetS) patients participating in this study.

	Women (*n* = 24)	Men (*n* = 26)	*p*
Age	55.0 (35.0−74.0)	56.0 (29.0−76.0)	0.736
BMI (kg/m^2^)	33.6 (30−48.6)	32.9 (30.4−45.3)	0.573
Drug treatments:			
AD-consumers	20 (40%)	22 (44%)	
LL-consumers	19 (38%)	20 (40%)	
HP-consumers	17 (34%)	19 (38%)	
Systolic BP (mm Hg)	136.0 (96.0−190.0)	134.0 (99.0−201.0)	0.165
Diastolic BP (mm Hg)	79.0 (55.0−102.0)	82.0 (60.0−96.0)	0.817
Tchol (mg/dL)	186.0 (116.0−249.0)	158.0 (106.0−269.0)	0.077
LDLc (mg/dL)	105.3 (49.0−163.0)	85.5 (46.0−163.0)	0.079
HDLc (mg/dL)	51.0 (28.5−85.5)	46.0 (30.0−65.0)	0.023 *
Triglycerides (mg/dL)	118.0 (48.0−479.0)	149.0 (64.0−611.0)	0.065
Glucose (mg/dL)	95.5 (79.0−194.0)	114.0 (73.0−232.0)	0.307
Insulin (μUl/mL)	9.7 (2.9−34.3)	15.9 (6.4−74.8)	0.027 *
HOMA-IR (U)	2.7 (0.9−9.3)	5.0 (1.4−15.1)	0.012 *
Bilirubin (U/L)	0.4 (0.2−0.7)	0.5 (0.2−1.1)	0.257
Protein (U/L)	7.0 (6.5−8.1)	7.2 (6.4−8.5)	0.682
GGT (U/L)	17.0 (8.0−147.0)	31.0 (12.0−283.0)	0.012 *
AST (U/L)	18.0 (12.0−43.0)	21.0 (11.0−64.0)	0.125
ALT (U/L)	19.0 (11.0−59.0)	23.0 (9.0−68.0)	0.105
ALP (U/L)	74.0 (44.0−279.0)	73.0 (38.0−317.0)	0.957
LDH (U/L)	175.0 (122.0−354.0)	179.0 (139.0−524.0)	0.511
Calcium (mg/dL)	9.5 (8.9−10.8)	9.6 (8.7−10.4)	0.920
Phosphorus (mg/dL)	3.5 (2.3−4.6)	3.3 (1.9−4.3)	0.174
Sodium (mEq/L)	141.0 (137.0−147.0)	141.0 (137.0−145.0)	0.444
Potassium (mEq/L)	4.5 (4.3−5.4)	4.5 (3.9−5.5)	0.260
Chlorine (mEq/L)	101.5 (95.0−105.0)	103.0 (99.0−105.0)	0.211
Albumin (g/dL)	4.5 (4.0−5.0)	4.6 (3.9−5.1)	0.687
Urea (mg/dL)	39.0 (23.0−87.0)	34.0 (22.0−88.0)	0.624
Creatinine (mg/dL)	0.7 (0.5−2.0)	0.9 (0.6−1.9)	0.024 *
LBP (µg/mL)	7.9 (7.2−9.2)	8.1 (7.1−10.4)	0.022 *
Acetic acid (µmol/L)	1249.6 (414.0−3432.4)	1697.1 (264.0−3373.2)	0.219
Propionic acid (µmol/L)	960.2 (292.5−3176.9)	1228.0 (167.9−2749.6)	0.135
Isobutyric acid (µmol/L)	127.5 (13.6−410.0)	167.9 (50.8−396.4)	0.180
Butyric acid (µmol/L)	1100.9 (241.5−3218.2)	1187.7 (148.1−3608.1)	0.204
Isovaleric acid (µmol/L)	180.7 (14.1−760.9)	264.1 (78.6−676.5)	0.210
Valeric acid (µmol/L)	194.7 (20.0−415.1)	284.2 (17.3−608.5)	0.166

Values are expressed as median and (range). BP, blood pressure; AD, oral anti-diabetic drugs; HP, anti-hypertensive drugs; LL, lipid-lowering drugs; GGT, gamma-glutamyl transferase; ALT, alanine aminotransferase; AST, aspartate aminotransferase, ALP, alkaline phosphatase, LDH, lactate dehydrogenase; Tchol, total cholesterol; LDLc, LDL cholesterol; HDLc, HDL cholesterol; LBP, lipopolysaccharide-binding protein. Significant differences (* *p* < 0.05) between men and women are highlighted in bold. The drug therapy is detailed in Table S2.

## Supplementary Materials

The following are available online at https://www.mdpi.com/2076-2607/8/3/416/s1, Figure S1: Comparison of the Chao-1 richness in OB, MetS (all), and according to their drug treatments (MetS-AD, MetS-LL, and MetS-HP). *Significantly different from the rest of the groups (*p* < 0.001). AD, oral anti-diabetics; LL, lipid-lowering drugs; HP, anti-hypertensive drugs. Figure S2: LEfSe analysis, performed on metabolic functions inferred by PICRUSt analysis, in **(a)** OB vs. MetS-all, **(b)**, OB vs. MetS-AD, **(c)** OB vs. MetS-LL, D) OB vs. MetS-HP, and within MetS patients, i.e., **(e)** MetS-AD vs. MetS non-AD, and **(f)** MetS-HP vs. MetS non-HP. No differences were found between MetS-LL and MetS non-LL (not shown). AD, oral anti-diabetics; LL, lipid-lowering drugs; HP, anti-hypertensive drugs. Figure S3: Comparison of fecal acetic and butyric acid concentrations in MetS-LL vs. MetS non-LL. LL, lipid-lowering drugs. Table S1: Demographic characteristics and plasma LBP values of obese volunteers. Table S2: Drug therapy followed by MetS patients in the present study.
