# Supplementary material for: There is No Distinctive Gut Microbiota Signature in the Metabolic Syndrome: Contribution of Cardiovascular Disease Risk Factors and Associated Medication
Source: Microorganisms. 2020 Mar 15;8(3):416. doi: 10.3390/microorganisms8030416 (PMC7143903; doi:10.3390/microorganisms8030416)
Supplement: Supplementary file 1 [file microorganisms-08-00416-s001.pdf]

microorganisms

Supplementary Materials

There is No Distinctive Gut Microbiota Signature in the Metabolic Syndrome: Contribution of Cardiovascular Disease Risk Factors and Associated Medication

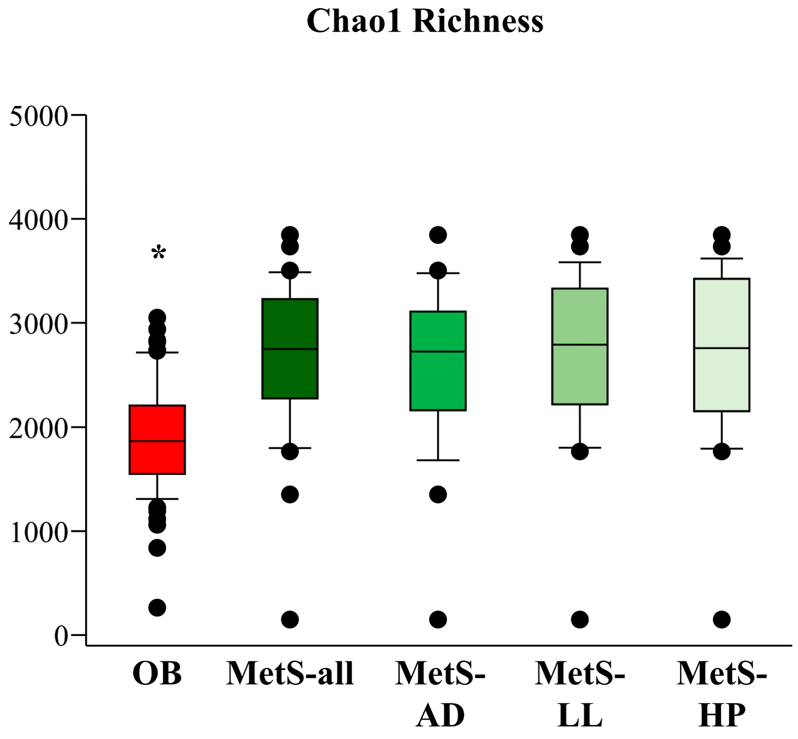

Supplementary Figure S1. Comparison of the Chao-1 richness in OB, MetS (all), and according to their drug treatments (MetS-AD, MetS-LL, and MetS-HP). *Significantly different from the rest of groups ($P < 0.001$). AD, oral anti-diabetics; LL, lipid-lowering drugs; HP, anti-hypertensive drugs.

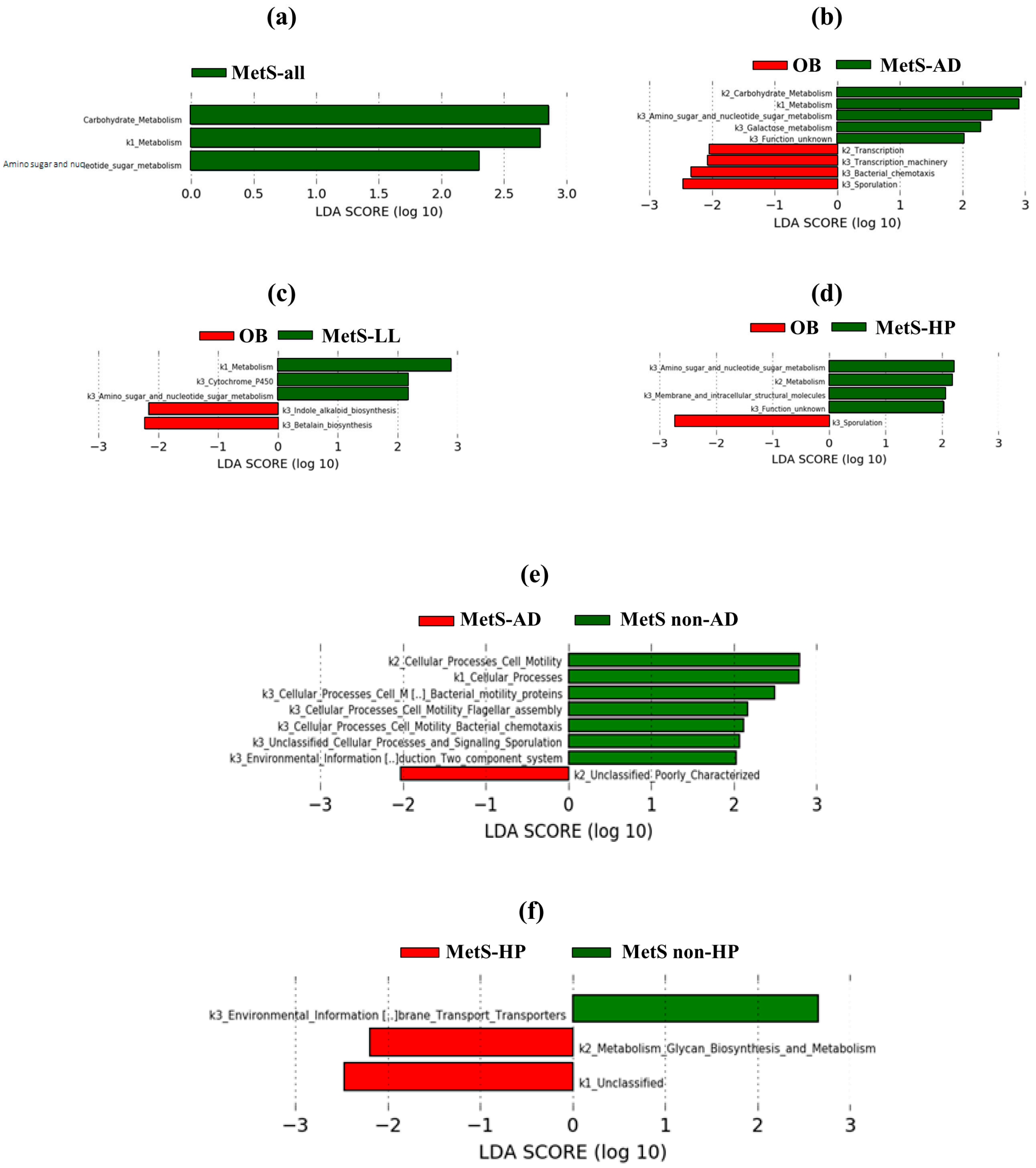

Supplementary Figure S2. LEfSe analysis, performed on metabolic functions inferred by PICRUSt analysis, in a) OB vs. MetS-all, b), OB vs. MetS-AD, c) OB vs. MetS-LL, d) OB vs. MetS-HP, and within MetS patients, i.e., e) MetS-AD vs. MetS non-AD, and f) MetS-HP vs. MetS non-HP. No differences were found between MetS-LL and MetS non-LL (not shown). AD, oral anti-diabetics; LL, lipid-lowering drugs; HP, anti-hypertensive drugs.

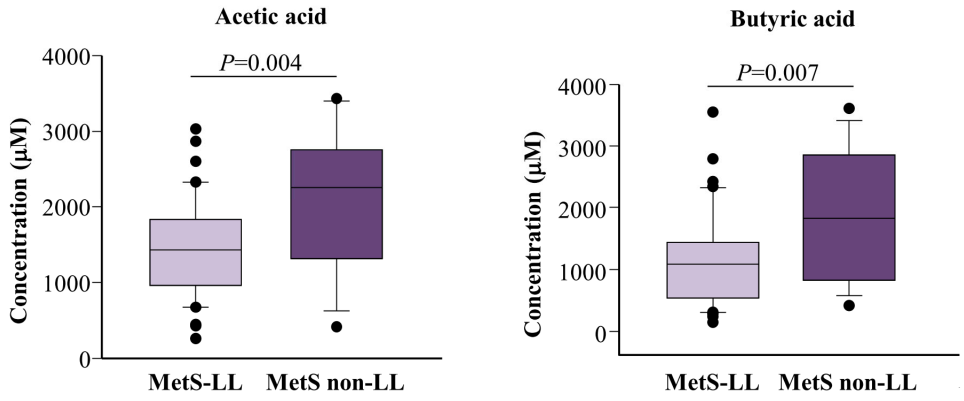

Supplementary Figure S3. Comparison of fecal acetic and butyric acids concentration in MetS-LL vs. MetS non-LL. LL, lipid-lowering drugs.

Table S1. Main demographic characteristics and plasma LBP values of obese volunteers*.

	Women (n= 37)	Men (n= 32)	*P*
Age	48.0 (34.0–61.0)	48.5 (31.0–69.0)	0.898
BMI (kg/m^2)	32.5 (30.0–43.3)	31.4 (30.1–37.1)	0.156
LBP (μg/mL)	7.8 (6.9–8.7)	7.7 (6.7–8.5)	0.569

*Serobiochemical variables were only available for a subsample of obese subjects (n=20) and are detailed below:

	Women (n=8)	Men (n=12)	*P*
Tchol (mg/dL)	208.5 (157.0-252.0)	207.0 (156.0-270.0)	0.649
LDLc (mg/dL)	143.5 (103.0-176.0)	158.0 (109.0-203.0)	0.251
HDLc (mg/dL)	59.5 (39.0-69.0)	45.5 (37.0-59.0)	**0.003***
Triglycerides (mg/dL)	101.0 (63.0-283.0)	104.5 (64.0-236.0)	0.728
Glucose (mg/dL)	87.5 (73.0-108.0)	92.5 (79.0-110.0)	0.249
Insulin (μUl/ml)	8.6 (6.7-14.1)	9.8 (3.8-19.6)	0.420
HOMA-IR (U)	2.1 (1.4-3.3)	2.4 (1.1-4.8)	0.440

Values are expressed as median and (range).

Table S2. Drug therapy followed by MetS patients in the present study.

Patient nº	Oral antidiabetic drugs (AD)	Lipid-lowering drugs (LL)	Anti-hypertensive drugs (HP)
1	Dapaglifosin, Metformin	Simvastatin	Valsartan, hydrochlorothiazide (HCT)
2	Albiglutide, Dapaglifosin, Metformin, Repaglinide	Simvastatin	Valsartan
3	Albiglutide, Metformin, Repaglinide	Pravastatin	HCT, Olmesartan
4	Dapaglifosin, Metformin	Atorvastatin	Eprosartan, HCT
5	Linagliptin, Metformin, Repaglinide	Simvastatin	Altizida, Amlodipine, Doxazosin, HCT, Olmesartan, Spironolactone
6	Empagliflozin, Lixisenatide, Metformin, Pioglitazone	Rosuvastatin	Olmesartan
7	Empagliflozin	Atorvastatin	Olmesartan
8	Metformin	Atorvastatin, Ezetimibe	Olmesartan
9	Dapaglifosin, Liraglutide, Metformin	Rosuvastatin	HCT, Irbesartan
10	Empagliflozin, Linagliptin, Metformin, Repaglinide	Atorvastatin	Amlodipine, HCT, Olmesartan
11	Exenatide, Metformin, Repaglinide	Rosuvastatin	Atenolol, Enalapril
12	Dapaglifosin, Metformin, Repaglinide, Sitagliptin	Simvastatin	Eprosartan, HCT
13	Dapaglifosin, Metformin	Atorvastatin	Amlodipine, Olmesartan
14	Canagliflozin, Metformin	Rosuvastatin	Amlodipine, Bisoprolol
15	Metformin	Ezetimibe	Enalapril
16	Alogliptin, Empagliflozin, Metformin, Pioglitazone	Rosuvastatin	Carvedilol, Ramipril
17	Liraglutide, Metformin, Pioglitazone	Atorvastatin	Telmisartan
18	Empagliflozin, Liraglutide, Metformin	Rosuvastatin	Amlodipine, Valsartan
19	Linagliptin	Rosuvastatin	Amlodipine, Bisoprolol, Furosemide, Telmisartan
20	Dapaglifosin, Metformin	Fibrate	Bisoprolol, HCT
21	Dapaglifosin, Liraglutide, Metformin	Simvastatin	Eprosartan, HCT
22	Dapaglifosin, Liraglutide, Metformin	Fibrate	Atenolol, Chlorthalidone
23	Dapaglifosin, Liraglutide, Metformin	Fibrate	Enalapril, Indapamide
24	Albiglutide, Empagliflozin	Rosuvastatin	Bisoprolol, Doxazosin
25	Dapaglifosin, Metformin	Atorvastatin	Olmesartan
26	Alogliptin, Metformin, Pioglitazone, Repaglinide	Atorvastatin	-
27	Dulaglutide, Metformin	Fibrate, Simvastatin	-
28	Alogliptin, Dapaglifosin, Exenatide, Metformin, Pioglitazone	Rosuvastatin	-
29	Canagliflozin, Metformin, Sitagliptin	Atorvastatin	-
30	Liraglutide, Metformin, Pioglitazone	Fibrate, Pravastatin	-
31	Dapaglifosin, Liraglutide, Metformin	Fibrate, Pravastatin	-
32	Metformin	-	Bisoprolol
33	Liraglutide, Metformin	-	Olmesartan
34	Metformin	-	Olmesartan
35	Dulaglutide	-	Enalapril, HCT
36	Dapaglifosin, Metformin	-	Enalapril
37	-	Atorvastatin	Amlodipine, Olmesartan
38	-	Pitavastatin	Candesartan
39	-	Fibrate, Pravastatin	Losartan
40	-	Simvastatin	Doxazosin, Manidipino, Olmesartan
41	-	Atorvastatin, Fibrate	Amlodipine, HCT, Olmesartan
42	-	Fibrate	HCT, Olmesartan
43	Liraglutide, Metformin	-	-
44	Liraglutide, Metformin	-	-
45	Empagliflozin	-	-
46	Dapaglifosin, Metformin	-	-
47	Liraglutide, Metformin	-	-
48	Empagliflozin, Liraglutide, Metformin	-	-
49	-	Pitavastatin	-
50	-	Fibrate	-

© 2020 by the authors. Licensee MDPI, Basel, Switzerland. This article is an open access article distributed under the terms and conditions of the Creative Commons Attribution (CC BY) license (http://creativecommons.org/licenses/by/4.0/).
